# Gender-Affirming Mastectomy in a Private Plastic Surgery Clinic in Poland: Sociodemographic Insights from a Cohort of 100 Transgender Individuals: A Retrospective Study

**DOI:** 10.3390/medicina61122148

**Published:** 2025-12-02

**Authors:** Klaudia Libondi, Guido Libondi, Wojciech M. Wysocki

**Affiliations:** 1 Uniestetica Center for Plastic and Reconstructive Surgery, 31-271 Krakow, Poland; 2 Maloposka Center for Plastic Surgery and Burns, Rydygier Hospital, 31-826 Krakow, Poland; 3 Faculty of Medicine and Health Sciences, Andrzej Frycz Modrzewski Krakow University, 30-705 Krakow, Poland; 4 Department of Oncological Surgery, 5th Military Clinical Hospital, 30-901 Krakow, Poland; 5 National Institute of Oncology, Maria Skłodowska-Curie Memorial, 02-781 Warsaw, Poland

**Keywords:** gender-affirming surgery, mastectomy, transgender individuals, chest wall reconstruction, healthcare in Poland

## Abstract

*Background and Objectives*: There is a worldwide increase in the demand for gender-affirming surgical treatments among transgender and gender-diverse (TGD) adults and adolescents. In Poland, transgender people generally lack trust in healthcare providers, which makes it more difficult for them to begin their transition process. This patient population is not well understood by many of the specialists who may potentially be involved in their care, in some way, reinforcing their concerns. The aim of this study is to present the sociodemographic characteristics of a group of female-to-male transgender patients who were admitted to a privately based plastic surgery center to undergo chest wall reconstruction. *Materials and Methods*: This study comprises a statistical analysis of data retrospectively obtained from the medical records of 100 patients from across the country undergoing female-to-male transition, who were operated on between 2021 and 2025 at a specialized private clinic in Poland. All individuals had already started gender-affirming medical treatment with testosterone at the time of first consultation. *Results*: The results show a trend toward a decreasing age at the time of the decision to undergo gender-affirming surgery. In the study group, 100% of patients were already undergoing hormone therapy. In our group of transgender individuals, we did not observe a correlation between cultural or social background, religion, and gender dysphoria. It is encouraging that more than half of the patients reported no longer needing psychiatric support, and that those who were still under specialist supervision stated that they experienced a significant improvement in their overall well-being. *Conclusions*: The rising demand for transgender healthcare highlights the need for studying and analyzing this group of patients in order to provide the best patient-centered care throughout the gender transition process by all specialists involved. Gender-affirming mastectomy, when combined with testosterone therapy, has a positive mental health impact on transgender individuals.

## 1. Introduction

The increasing recognition of the need for adequate healthcare services for transgender individuals is clearly evident. Our data reflect the global trend, which is likely a result of increased societal acceptance of gender diversity, greater tolerance, and the visibility of transgender individuals both in real life and in the media [1,2]. Therefore, it is crucial for doctors providing care to transgender patients to thoroughly document the entire process of transition. Gender dysphoria is characterized by a marked and persistent incongruence between an individual’s experienced or expressed gender and the sex assigned to them at birth.

The father of transgender healthcare, endocrinologist Harry Benjamin, developed diagnostic criteria for the qualification of patients for gender confirmation treatment [3]. This is known as the classical approach, according to which patients must meet certain criteria, such as an early onset of dysphoria, a binary gender definition, or passing the traditional “real-life test” by at least two years.

The Polish Society of Sexology published its recommendations on medical care for transgender adults in 2021 [4] as guidance for healthcare professionals in supporting individuals with gender incongruence throughout the transition process, from diagnosis to medical and surgical procedures.

Gender-affirming mastectomy (GAM) is the most common reconstructive procedure performed in female-to-male (FtM) patients. It is often the first and only gender-affirming surgical treatment (GAST), with a high satisfaction rate [5,6,7,8,9] and a regret rate of less than 1% [10].

In Poland, a recent study [11] highlighted that transgender individuals identify a fear of discrimination as the primary factor preventing them from accessing medical services. Other contributing factors include anxiety about potential reactions from their social environment, the perceived lack of knowledge among medical personnel regarding the specific healthcare needs of transgender patients, and a general lack of trust toward medical staff. Fewer than two percent of respondents indicated a lack of financial resources as a barrier.

It is, therefore, of paramount importance to reverse the prevailing trend of distrust that transgender individuals exhibit toward medical professionals. Such a shift can only be accomplished through a deeper and more comprehensive understanding of this specific patient population, accompanied by the rejection of stereotypes that hinder the establishment of a trusting doctor–patient relationship.

This retrospective study analyses the sociodemographic and clinical characteristics of 100 consecutive FtM patients in Poland treated at a single private specialized center.

## 2. Methods

In the setting of a private surgical clinic, we consult FtM patients during the transition process. At the time of the surgical consultation, the patient is already undergoing gender-affirming hormone treatment for at least six months. The surgical procedure is tailored to the patient, taking into account their anatomical characteristics and personal preferences. Three months prior to the reconstructive procedure, a letter from one of the mental healthcare professionals on the multidisciplinary team is requested.

A retrospective audit of electronic medical data was conducted on a group of 100 consecutive transgender individuals who underwent surgery at a private plastic surgery practice in Kraków, Poland. Electronic medical data are accessible only to the authors and the patients’ consultants. The database was prepared by the two authors (K.L. and G.L.), and a postoperative telephone interview was conducted in a private office by one of the patient’s consultants to assess whether the patient had interrupted psychiatric treatment or was continuing it. To evaluate the influence of GAST on the patient’s overall psychological well-being, consultants asked how the recent GAM had improved their lives. Patients responded using a numerical rating scale (NRS) from 0 to 3, where 0 indicated “not at all”, 1 indicated “not much”, 2 indicated “moderately”, and 3 indicated “significantly”.

All individuals had already started gender-affirming medical treatment with testosterone at the time of the first consultation and were consulted and operated on by the plastic surgery specialists team between May 2021 and June 2025. A minimum of six months of testosterone administration was required before undergoing chest wall surgical masculinization.

The sociodemographic data included in the analysis are as follows: age at first consultation, age on the day of surgery, changes in demographic data at the time of the operation, level of education of both the patients and their parents, religious beliefs, and urban/rural residency. Clinical data included psychiatric comorbidities.

### Statistical Analysis

The analysis of qualitative variables (i.e., those not expressed numerically) was performed by calculating the absolute and percentage frequencies of all possible values these variables could take.

Comparisons of qualitative variable values between groups were conducted using the chi-squared test (with Yates’ correction for 2 × 2 tables) or Fisher’s exact test when the assumptions of the chi-squared test regarding expected frequencies were not met.

A significance level of 0.05 was adopted for the analysis, and all *p*-values below 0.05 were considered to indicate significant associations.

The analysis was performed using R software, version 4.5.0.

## 3. Results

Data were collected for a total of 100 consecutive individuals who underwent surgery at a private plastic surgery clinic over a period of four years and one month, under the care of two plastic surgery specialists. In 2021, six patients were operated on; in 2022, 17 patients; in 2023, 28 patients; in 2024, 28 patients; and during the first half of 2025, 21 patients.

All of our patients identified as trans-male. On the day of the visit, 28% of patients had already officially changed their personal data and updated their documents (Female to Male).

The median age of patients was 23.1 years (range: 16–41 years). Seventeen percent of patients (*n* = 17) were under the age of 18 at the time of their first visit, while only 5% (*n* = 5) were minors at the time of surgery. Of these, two patients were 16 years old, and three were 17 years old. This reflects the authors’ policy of postponing surgical intervention until the patient reaches the age of 18 and is able to provide informed consent independently. Exceptions to this policy were made only in cases of pronounced gender dysphoria, combined with confirmed acceptance and support from both parents.

In terms of age distribution, 32% of patients were between 18 and 20 years old, and 54% were in the 21–30 age group. Only 9% of individuals undergoing mastectomy were older than 31 years.

In the analyzed cohort, 45% of patients (*n* = 45) resided in cities with populations exceeding 500,000. Interestingly, 36% (*n* = 36) originated from small towns with fewer than 50,000 inhabitants. A further 12% (*n* = 12) lived in cities with populations between 100,000 and 200,000, while 6% (*n* = 6) were residents of cities with populations ranging from 200,000 to 500,000.

Statistical analysis revealed no significant association between the occurrence of gender dysphoria and the size of the urban area of residence (i.e., number of inhabitants in the hometown). Moreover, no evidence was found of a relationship between the presence of any mental health disorders and the population size of the place of residence in the studied group (Table 1).

At the time of surgery, no statistically significant differences were observed between patients who had already legally changed their identity data and those who had not, with respect to the population size of their city of origin (Table 2).

Regarding educational attainment, 71% of patients (*n* = 71) reported having completed secondary education, a proportion consistent with the age distribution of the main cohort treated at our clinic. Twenty-seven percent (*n* = 27) held a university degree, while 2% (*n* = 2) had not graduated from secondary school.

Among the parents, 60% of mothers and 54.5% of fathers held a university degree, and 38.4% of mothers and 39.4% of fathers were secondary school graduates.

No statistically significant correlation was identified between parental educational level and the presence of concomitant psychiatric disorders in patients (Table 3).

### 3.1. Religion and Dysphoria

Eighty-one percent of patients (*n* = 81) did not identify with any religion and described themselves as non-believers, while 19% (*n* = 19) identified as having faith or religious affiliation (believers).

Analysis of the relationship between religious identification and the presence of psychiatric disorders revealed no statistically significant difference between believers and non-believers (*p* > 0.05; Table 1). Among believers, 62.69% did not present with concomitant mental health disorders, compared to 52.63% of non-believers (*p* = 0.569).

### 3.2. Psychiatric Comorbidities

Thirty-seven percent of patients (*n* = 37) were diagnosed with a psychiatric condition requiring ongoing psychiatric care. The most common disorder was depression, affecting 70.2% (*n* = 26) of these patients, followed by anxiety, observed in 18.9% (*n* = 7), and attention-deficit/hyperactivity disorder (ADHD), present in 10.8% (*n* = 4). Additionally, 8.1% (*n* = 3) of patients had at least two of the aforementioned psychiatric conditions.

No statistically significant association was found between the presence of psychiatric disorders and the patient’s age at the time of the initial consultation.

At a mean follow-up of 16 months (range 3–45 months) after surgery, 56.7% (*n* = 21) of patients were no longer under psychiatric care, while the remaining 16 patients (43.3%) continued psychiatric supervision. Of these 16 patients, 93.7% (*n* = 15) self-reported a significant improvement in overall well-being (3/3 on the NRS 0–3 scale), and 6.3% (*n* = 1) reported a moderate improvement (2/3 on the NRS 0–3 scale).

## 4. Discussion

There is a worldwide increase in the demand for gender-affirming medical (GAMT) and surgical treatments (GAST) among the transgender and gender-diverse (TGD) adult and adolescent population [12]. This trend aligns with the ongoing demedicalization of approaches to gender dysphoria. Gender specifically refers to the social construct created by society that gives the individual a certain set of rules to follow in order to be fully accepted as that gender. Sex, on the other hand, refers to the biological component where an individual is assigned male or female based on their sexual organs at birth [13,14].

In the eleventh version of the International Classification of Diseases (ICD-11), which came into effect in 2022, two entities were introduced: gender incongruence of adolescence or adulthood and gender incongruence of childhood [15] (“Gender incongruence of adolescence and adulthood is characterized by a marked and persistent incongruence between an individual’s experienced gender and assigned sex, which often leads to a desire to ‘transition’ in order to live and be accepted as a person of the experienced gender. This may involve hormonal treatment, surgery, or other healthcare services aimed at aligning the individual’s body, insofar as possible, with the experienced gender. The diagnosis cannot be assigned prior to the onset of puberty” [15].

Health professionals also use the 5th edition of the Diagnostic and Statistical Manual of Mental Disorders (DSM-V), released in 2015, to diagnose gender incongruence [16]. Gender dysphoria is defined as “a marked incongruence between one’s experienced/expressed gender and assigned gender, of at least six months’ duration, as manifested by at least two or more of the following: ….” The DSM-V includes a separate diagnostic classification for children and allows this diagnosis to be applied to individuals with disorders of sex development (DSD).

Both the DSM-V (earlier) and ICD-11 (later) reflect the modern understanding of sexual health and gender identity. These changes aim to move beyond older concepts of mental illness and reduce the social stigma historically associated with gender incongruence.

The Polish Sexuology Society published recommendations on medical care for transgender adults in 2021 [4], providing guidance for health professionals in supporting individuals with gender incongruence throughout the transition process—from diagnosis to medical and surgical treatment. These recommendations are consistent with the seventh edition of the Standards of Care (SOC-7) published by the World Professional Association for Transgender Health (WPATH) in 2012. Following a diagnosis of gender dysphoria (DSM-V), a transgender or gender-diverse (TGD) adult may seek gender-affirming medical and/or surgical treatments (GAMSTs).

Gender-affirming mastectomy (GAM) is a surgical intervention that catalyzes a symbolic and emotional realignment between body image and internal identity, leading to a more stable, integrated masculine self-perception. The surgery functioned not merely as a physical correction but as a therapeutic turning point [17].

In this article, we analyzed data from a defined population of TGD adults and adolescents. One hundred trans men who underwent gender-affirming mastectomy (GAM) had already been receiving gender-affirming medical treatment (GAMT) with hormone therapy for at least six months before surgery. Data were collected at a single private plastic surgery center in Cracow, Poland, over a period of four and a half years. In Poland, gender-affirming surgery is not reimbursed by the National Health Fund.

When compared with international and national studies [12,18], our sample represents a comparable cohort size. Moreover, considering the time span (a total of 4 years and 1 month), we conclude that our center manages a high caseload of gender-affirming surgeries. This represents one of the largest patient cohorts in Poland treated at a single institution.

The demand for gender-affirming surgery increased nearly fivefold in the United States between 2016 and 2021 [12]. While the mean age of patients undergoing GAM was approximately 30 years in Central and Western Europe between 1990 and 2014 [6] and 28 years in the largest Polish study (1991–2006) [18], the mean age in our cohort was 23 years. Only 5 patients were underage at the time of surgery: 3 were 17 years old, and 2 were 16. Seventeen patients were underage at the time of their first consultation. This partly reflects the one-year average waiting time between consultation and surgery, and partly our preference to postpone surgery until the patient reaches the age of 18. The authors proceeded with surgery in the 5 underage patients because of the strong family support demonstrated during the initial consultation. The support has been shown to provide positive mental health outcomes and well-being for minors [19].

In Poland, from the age of 16, legally informed consent must be signed both by the patient and their parents. From an ethical point of view, while supporting the autonomy of the minors, the authority of making a decision is shared with both parents.

In two cases, only one parent attended the first consultation: in one case, the mother attended while the father opposed the decision; in the other, the father no longer resided in Poland. In the first case, surgery was postponed until the patient turned 18. In the second, a notarized statement from the absent father validated the consent.

In our clinic, we adhere to the guidelines of the Standard of Care of the WPATH and the Polish Sexuology Society: a patient may be considered eligible for treatment only after a qualified mental health professional provides a recommendation letter that confirms a patient is ready for gender affirming surgery.

In the largest retrospective cohort study published until now, between 2013 and 2020, a 3-fold increase in the incidence of gender affirming mastectomy in the adolescent population in the USA was found. Among 249 adolescents, no patients underwent reversal, only two expressed regrets, and the prevalence of surgical complications was low [20]. These data are particularly important for the discussion of ethical considerations, especially with regard to concerns related to beneficence and non-maleficence in treatment

The authors decided to include only transgender men who had already received gender-affirming medical treatment (testosterone) for at least six months prior to surgery. This reflects a conservative and safety-oriented approach to a relatively novel patient group.

While the positive effects of gender-affirming medical and surgical treatments (GAMSTs) on mental health, quality of life, and body comfort are well established, the surgical experiences of nonbinary patients remain insufficiently investigated [21].

Individual mental health outcomes are substantially shaped by the interaction of socioeconomic status, physical environment, social support, and healthcare access [22]. Gender incongruence has a neurobiological basis, but it is closely associated with the individual’s interaction with the external world, their self-perception and the feedback received in return [23]. In our study, no relationship was observed between urban versus rural residence and the incidence of gender dysphoria or related mental health disorders. Gender dysphoria is not confined to urban settings, nor is it the result of provincial or conservative attitudes in rural settings. Similarly, no association was found between residence type and legal recognition: rural and urban patients were equally likely to have changed their identity documents by the time of surgery. Poland ranks among the lowest in Europe in terms of acceptance and legal protection of LGBT+ rights [24]. However, findings from a large-scale survey conducted across 28 European countries by the EU Agency for Fundamental Rights indicate that even in the least conservative countries—such as Sweden and Finland—nearly 40% of respondents reported experiencing discrimination and harassment due to being LGBT [25].

During the penultimate administration, led by a coalition of right-wing conservative parties, the socio-political climate remained markedly hostile, characterized by institutionalized discrimination and political rhetoric framing LGBT+ identities as threats to national and religious values [26]. Nevertheless, the political realignment resulting from the most recent elections has initiated a discernible shift in policy orientation toward transgender individuals.

Until 2025, transgender individuals in Poland seeking to change their legal gender were required to undergo a lengthy and complex court process, often involving litigation against their parents. A prerequisite was evidence of having already initiated medical transition (GAMT). In 2025, however, a new law simplified the process by replacing litigation with a straightforward application procedure [27]. Under this law, applicants submit documentation from a psychologist, a psychiatrist, or a sexologist confirming gender incongruence. Neither GAMT nor GAST is required. This reform markedly reduced arbitrariness, shortened timelines, and removed dependence on the subjective interpretation of individual judges. It also alleviated barriers posed by costs and rural residence.

Regarding educational attainment, 27% of patients hold a university degree. Since 63% of our patients were over 21 years old, it can be assumed that nearly half of those within the typical age for higher education had completed a university program. Parental education showed no significant correlation with patients’ psychiatric comorbidities. More than half of our patients had at least one parent with higher education, with mothers more often than fathers holding a degree.

Poland, a country with strong Christian Catholic traditions, has experienced a decline in religious affiliation and practice since the fall of communism. Consistent with this trend, 81% of the study population reported no religious identification. Among the 19 patients who declared religious belief, only two identified as Catholic.

No correlation was found between religious identification and psychiatric comorbidities. This suggests that psychiatric conditions in transgender individuals are neither explained by the absence of religious belief nor by religious indoctrination.

When analyzing psychiatric comorbidities, depression was the most common disorder, affecting 26% of all patients and accounting for 70.2% of those with a psychiatric diagnosis. The prevalence of depression in our cohort was nearly half that reported in both international and national studies [11,28]. In line with the literature, no patients were found to suffer from severe psychiatric disorders [29].

One of the most striking findings was that more than half of patients (51%) did not require ongoing psychiatric care after surgery. Moreover, a clear improvement in psychological well-being was noted in those who remained under psychiatric care, with many reporting an NRS score of 3, indicating significant positive change. These findings align with recent literature demonstrating the combined benefit of gender-affirming surgery (GAST) and medical treatment (GAMT) on quality of life, body image, and psychopathology in adults with gender dysphoria [30].

### Limitations

There are a number of limitations to this cross-sectional survey.

In addition to the limitations inherent in the study design, including the absence of a control group, data were collected from a single private center in Cracow, the second-largest city in Poland. Some doubts may arise regarding patients treated at other centers; however, as our patients are geographically dispersed across the country, it is likely that similar findings would be observed elsewhere; however, we are uncertain of referral patterns in other centers. Data on overall well-being were collected through a self-reported assessment conducted via telephone interview. The suitability of telephone interviews for addressing potentially sensitive topics, such as mental health, has been previously described [31,32]. Moreover, from a patient’s perspective, telephone calls are cost-effective and less time-consuming, especially given that patients are geographically dispersed across the country. The question of whether a patient continues psychiatric therapy was intended to determine whether individuals who had previously been under psychiatric care still required treatment after surgery and medical therapy. A response of “I am no longer under treatment” indicates that, at the time of questioning, the patient feels recovered from the psychiatric conditions associated with untreated gender dysphoria, suggesting that the transition process itself has had a positive effect. For patients who responded, “I am still under treatment”, their consultant asked them to quantify how the surgery had improved their overall well-being. The use of a four-point rating scale can be considered a methodological weakness; however, to a direct question, there can only be an equally direct answer. It was not the authors’ intention, nor the aim of this work, to investigate the psychological aspect in depth. Our results should be accepted with care. This topic is addressed in other studies cited within our paper, which serve as a foundation for constructing a more streamlined discussion aimed at a broader audience of professionals. In the near future, we intend to explore the topic in greater depth using the TRANS-questionnaire (TRANS-Q), a novel validated pre- and post-operative satisfaction tool in patients undergoing gender confirming mastectomy [33]. Given the aforementioned limitations, our results should be accepted with care.

## 5. Conclusions

In Poland, there is a growing trend of trans-male individuals seeking gender-affirming surgery at increasingly younger ages. Gender dysphoria is frequently associated with high rates of preoperative psychiatric comorbidities, particularly depression. However, no significant correlations were found between mental health status and demographic, social, or geographic factors. Postoperative follow-up revealed substantial psychological improvement in the majority of patients, highlighting the positive mental health impact of gender-affirming surgery for this population. Among patients receiving treatment for psychiatric comorbidities, more than half reported that they were no longer undergoing psychiatric therapy. Among those who were still in treatment, gender-affirming mastectomy significantly improved overall well-being in the vast majority of patients.

This study is particularly relevant for specialists who, being unfamiliar with this specific group of patients, wish to gain a deeper understanding of their background beyond preconceived notions.

## Figures and Tables

**Table 1:** Size of urban area of origin and patient’s psychiatric disorder.

Psychiatric Disorder	City of Residency, Number of Inhabitants				*p*
	up to 50,000 (*n* = 36)	50–200,000 (*n* = 12)	200–500,000 (*n* = 6)	More than 500,000 (*n* = 45)	
None	18 (50.00%)	9 (75.00%)	4 (66.67%)	29 (64.44%)	*p* = 0.393
Depression	11 (30.56%)	2 (16.67%)	2 (33.33%)	11 (24.44%)	*p* = 0.74
Anxiety disorder	4 (11.11%)	1 (8.33%)	1 (16.67%)	3 (6.67%)	*p* = 0.68
ADHD	1 (2.78%)	0 (0.00%)	0 (0.00%)	4 (8.89%)	*p* = 0.586

*p*—test chi-squared or exact Fisher test.

**Table 2:** Size of urban area of origin and legal recognition of gender reassignment at the moment of the operation.

Changed Personal Documents	City of Residency, Number of Inhabitants				*p*
	up to 50,000 (*n* = 36)	50–200,000 (*n* = 12)	200–500,000 (*n* = 6)	More than 500,000 (*n* = 45)	
Yes	12 (33.33%)	4 (33.33%)	1 (16.67%)	10 (22.22%)	*p* = 0.641
No	24 (66.67%)	8 (66.67%)	5 (83.33%)	35 (77.78%)	

*p*—exact Fisher test.

**Table 3:** Higher educational level among parents and psychiatric disorders.

Psychiatric Disorder	Level of Education of Parents		*p*
	Middle (*n* = 32)	High (*n* = 63)	
None	22 (68.75%)	36 (57.14%)	*p* = 0.382
Depression	5 (15.62%)	19 (30.16%)	*p* = 0.197
Anxiety disorder	5 (15.62%)	3 (4.76%)	*p* = 0.114
ADHD	1 (3.12%)	4 (6.35%)	*p* = 0.66

*p*—test chi-squared or exact Fisher test.

## Data Availability

The original contributions presented in this study are included in the article. Further inquiries can be directed to the corresponding author.
